# Do the Best Teachers Get the Best Ratings?

**DOI:** 10.3389/fpsyg.2016.00570

**Published:** 2016-04-25

**Authors:** Nate Kornell, Hannah Hausman

**Affiliations:** Department of Psychology, Williams College, Williamstown, MAUSA

**Keywords:** student evaluations of teaching, teacher ratings, long-term learning, grades, ratings

## Abstract

We review recent studies that asked: do college students learn relatively more from teachers whom they rate highly on student evaluation forms? Recent studies measured learning at two-time points. When learning was measured with a test at the end of the course, the teachers who got the highest ratings were the ones who contributed the most to learning. But when learning was measured as performance in subsequent related courses, the teachers who had received relatively low ratings appeared to have been most effective. We speculate about why these effects occurred: making a course difficult in productive ways may decrease ratings but enhance learning. Despite their limitations, we do not suggest abandoning student ratings, but do recommend that student evaluation scores should not be the sole basis for evaluating college teaching and they should be recognized for what they are.

## Do the Best Teachers Get the Best Ratings?

Calvin: “Here’s the latest poll on your performance as dad. Your approval rating is pretty low, I’m afraid.” Dad: “That’s because there’s not necessarily any connection between what’s good and what’s popular. I do what’s right, not what gets approval.” Calvin: “You’ll never keep the job with that attitude.” Dad: “If someone else offers to do it, let me know.”

–Calvin and Hobbes, Bill Watterson, February 13, 1994

Student evaluations of teaching are one of the main tools to evaluate college teaching (Clayson, 2009; Miller and Seldin, 2014). Ratings of factors like clarity, organization, and overall quality influence promotion, pay raises and tenure in higher education. Thus, we asked: Do better teachers get better ratings? Being a “better teacher” can be defined many ways, such as teaching that inspires students to work hard or get interested in a subject, but in this article we equate good teaching with meaningful student learning. Therefore, our question is, do students give the highest ratings to the teachers from whom they learn the most? Given the ubiquity and importance of teacher ratings in higher education, we limited our review to research conducted with college students.

## A Framework for Understanding Teacher Ratings

**FIGURE 1:**
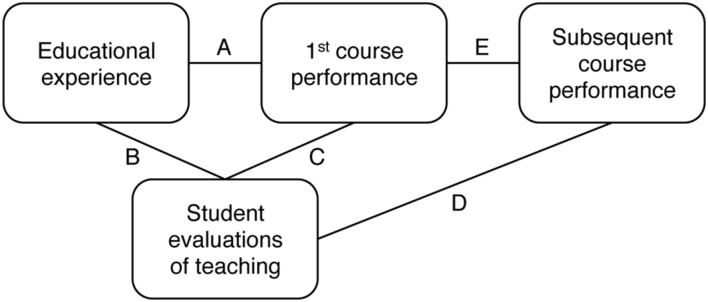
**Framework for understanding possible influences on student evaluations of teaching**.

**Figure 1** presents a framework for understanding teacher ratings. This framework is simply a way of organizing the possible relationships among what students experience in a course, the ratings they give their instructor, and how much they learn. In this article, “ratings” refers to students’ responses to a single survey question about overall instructor quality. While students also typically rate instructors on preparedness, content knowledge, enthusiasm, clarity of lectures, etc., responses to these questions were not the primary focus of the studies we reviewed.

In the figure, educational experience is the broad term we are using to refer to everything students experience in connection with the course they are evaluating (e.g., teacher age, gender, and charisma, topic of the course, font used on class handouts, and temperature in the classroom). The first course is the one taught by the professor being evaluated. Performance in the first course reflects students’ knowledge of the information that course was designed to teach. Subsequent course performance means how those same students do in related, follow-up courses. Subsequent course performance is included because, for example, a good Calculus I teacher should have students who do relatively well in follow-up courses that rely on calculus knowledge, like Calculus II and engineering. Our main interest was the relationship between how college students evaluate an instructor and how much they learn from that instructor, which is represented by the C and D links in **Figure 1**.

## Educational Experience and Ratings

Some links in **Figure 1** have been researched more extensively than others. “Literally thousands” (Marsh, 2007, p. 320) of articles have examined the relationship between educational experience and teacher ratings—that is, the B link in **Figure 1**. They have identified an extensive list of student, instructor, and course characteristics that can influence ratings, including student gender, prior subject interest, and expectations for the course; instructor gender, ethnicity, attractiveness, charisma, rank, and experience; and course subject area, level, and workload (for reviews, see Neath, 1996; Marsh and Roche, 1997; Wachtel, 1998; Kulik, 2001; Feldman, 2007; Pounder, 2007; Benton and Cashin, 2012; Spooren et al., 2013).

This literature is difficult to succinctly review because the results are so mixed. For many of the questions one can ask, it is possible to find two articles that arrive at opposite answers. For example, a recent randomized controlled experiment found that students gave online instructors who were supposedly male higher ratings than instructors who were supposedly female, regardless of their actual gender (MacNell et al., 2014). On the other hand, Aleamoni (1999) referred to the effect of instructor gender on teacher ratings as a “myth” (p. 156). Other studies suggest that the relationship between a teacher’s gender and ratings may depend on the student’s gender as well as whether the teacher’s behavior conforms to gender stereotypes (for a review see Pounder, 2007; e.g., Boring, 2015). One reason studies come to such different conclusions may be the fact that many studies do not exercise high levels of experimental control: They do not experimentally manipulate the variable of interest or do not control for other confounding variables. But variable results may also be inherent in effects of variables like instructor gender, which might not be the same for all types of students, professors, subjects, and course levels. Finally, the mixed results in this literature may be due to variability in how different teacher evaluation surveys are designed (e.g., negatively worded questions, number of response options, and neutral response options) and administered (e.g., was the teacher present, was a tough assignment handed back just prior, did it take place online, were there incentives for filling it out; Wachtel, 1998; Spooren et al., 2013; Stark and Freishtat, 2014).

The point is it is difficult to draw general conclusions from existing research on the relationship between teacher ratings and students’ educational experiences. Our goal is not to review this literature in detail, but to discuss what it means for the question of whether better teachers get higher ratings. The educational experience variables that affect ratings can be classified into two categories: those that also affect learning and those that do not. Presumably, instructor attractiveness and ethnicity should not be related to how much students learn. Instructor experience could be however. Instructors who have taught for a few years might give clearer lectures and assign homework that helps students learn more than instructors who have never taught before (McPherson, 2006; Pounder, 2007). If teacher ratings are mostly affected by educational experience variables that are not related to learning, like instructor attractiveness and ethnicity presumably, then teacher ratings are not a fair way to identify the best teachers. It is possible though that teacher ratings primarily reflect student learning, even if some variables like attractiveness and ethnicity also affect ratings, but to a much smaller degree. However, most of the studies covered in the reviews of the B link do not measure student learning objectively, if at all. Therefore, the studies identify educational experience factors that affect ratings, but do not shed light on whether students give higher ratings to teachers from whom they learn the most. Thus, they are not directly relevant to the present article.

## Features of the Ideal Study of Ratings and Student Learning

To answer our main question—whether teachers with higher ratings engender more learning (i.e., the C and D links in **Figure 1**)—a study would, ideally, have all of the characteristics described in **Table 1**. These features describe what a randomized controlled experiment on the relationship between ratings and learning would look like in an educational setting.

**Table 1:** Ideal features of a study that measures the relationship between ratings and learning.

Evaluations are actual ratings obtained by a college or university (i.e., not data from a lab study).
Related subsequent courses are required.
Students are assigned to instructors randomly for the first course and subsequent courses.
The same (or comparable) objective measures of student knowledge are used for all instructors teaching a given course.

The features in **Table 1** are desirable for the following reasons. First, a lab study cannot simulate spending a semester with a professor. Second, if the subsequent courses are not required, the interpretation of the results could be obscured by differential dropout rates. For example, a particular teacher would appear more effective if only his best students took follow-up courses. Third, random assignment is necessary or else preexisting student characteristics could differ across groups—for example, students with low GPAs might gravitate toward teachers with reputations for being easy. Fourth, comparable (or identical) measures of student knowledge allow for a fair comparison of instructors. (Course grades are not a valid measure of learning because teachers write their own exams and the exams differ from course to course.) Next, we review the relationship between ratings and first course performance (i.e., in the professor’s own course). Then we turn to newer literature on the relationship between teacher ratings and subsequent course performance.

## Teacher Ratings and First Course Performance

A wealth of research has examined the relationship between how much students learn in a course and the ratings they give their instructors (i.e., the C link in **Figure 1**). This research has been synthesized in numerous reviews (Abrami et al., 1990; Cashin, 1995; Kulik, 2001; Feldman, 2007; Marsh, 2007) and meta-analyses (Cohen, 1981, 1983; Dowell and Neal, 1982; McCallum, 1984; Feldman, 1989; Clayson, 2009). The studies included in these meta-analyses had the following basic design: Students took a course with multiple sections and multiple instructors. Objective measures of knowledge (e.g., a common final exam or essay) and teacher evaluations were administered at the end of the course.

The evidence from all of the meta-analyses suggests that there is a small positive relationship between ratings and first course performance: better teachers, as measured by the average of their students’ end-of-semester performance, do get slightly higher average ratings. **Table 2** shows the mean correlation between an overall measure of teacher effectiveness and first course performance. Cohen (1981) reported the highest average correlation of 0.43 with a 95% confidence interval ranging from 0.21 to 0.61. This means that teacher ratings account for only about 18% of the variability in how much students learn, at best. Clayson (2009) reported the lowest mean correlation of 0.13 with a standard error of 0.19, concluding the correlation between ratings and first course performance is not significantly different from zero. **Table 2** also shows that first course performance was positively correlated with other evaluation questions as well, such as ratings of the instructor’s preparation, the organization of the course material, and how much students thought they had learned. The studies in **Table 2**, and the studies described in the sections that follow, did not examine individual students’ ratings and performance; they measured something more interesting for present purposes: the correlation at the course-section level between an instructor’s mean ratings and his or her section’s mean test scores. (For a technical take on why and how to aggregate individual student ratings at the course-section level to estimate teacher effectiveness, see Lüdtke et al., 2009; Marsh et al., 2012; Scherer and Gustafsson, 2015).

**Table 2:** Mean correlations between ratings and first course performance.

Meta-analysis	Overall	Instructor	Course	Perceived
	effectiveness	preparation	organization	learning
Clayson (2009)	0.13^a^	–	–	–
Cohen (1981)	0.43	0.50	0.47	0.47
Cohen (1983)	0.38	–	–	0.47
Dowell and Neal (1982)	0.20	–	–	–
Feldman (1989)	–	0.57	0.56	0.46
McCallum (1984)	0.32	–	–	–

*^a^Not significantly different from 0. All other correlations are significant at the 0.05-level.*

## Teacher Ratings and Subsequent Course Performance

A few recent studies have examined the relationship between ratings, first course performance, and crucially, subsequent course performance, which has been advocated as a measure of long-term learning (Johnson, 2003; Yunker and Yunker, 2003; Clayson, 2009; Weinberg et al., 2009; Carrell and West, 2010; Braga et al., 2014). Subsequent-related course performance is arguably more important than first course performance because the long-term goal of education is for students to be able to make use of knowledge after a course is over.

It is important to distinguish between student knowledge and teacher contribution to student knowledge. Students who do well in the first course will tend to do well in subsequent related courses (e.g., Hahn and Polik, 2004; Lee and Lee, 2009; Kim et al., 2012), but raw student performance is not the issue at hand when evaluating teacher effectiveness. The issue is how much the teacher contributes to the student’s knowledge. The studies we describe next used value-added measures to estimate teacher contribution to knowledge.

Since there is typically a positive relationship between ratings and first course performance, we might also predict a positive relationship between ratings and subsequent performance. Yet, three recent studies suggest that ratings do not predict subsequent course performance (Johnson, 2003; Yunker and Yunker, 2003; Weinberg et al., 2009). These studies represent an important step forward, but they are open to subject-selection effects because students were not assigned to teachers randomly and follow-up courses were not required; additionally, only Yunker and Yunker (2003) used an objective measure of learning (a common final exam).

Only two studies, conducted by Carrell and West (2010) and Braga et al. (2014), have all of the characteristics in **Table 1**. We review these studies next. Carrell and West (2010) examined data collected over a 10-year period from over 10,000 students at the United States Air Force Academy. This dataset has many virtues. There was an objective measure of learning because students enrolled in different sections of a course took the same exam. (The professors could see the exams before they were administered.) Lenient grading was not a factor because each professor graded test questions for every student enrolled in the course. Students were randomly assigned to professors. Finally, the effectiveness of the nearly 100 Calculus I instructors was measured in two ways, once based on their students’ grades in Calculus I and once based on their students’ grades in related follow-up courses. The concern that only the best students in certain professors’ courses chose to take a follow-up course was eliminated because follow-up courses were mandatory.

Carrell and West (2010) used value-added scores to measure teacher effectiveness. For each student in a particular Calculus I section, the student’s characteristics (e.g., incoming test scores) and characteristics of the class (e.g., class size) were used to predict the student’s grade. The predicted grade was compared to the student’s actual grade. The difference between the actual and predicted grade can be attributed to the effect of the teacher, since non-teacher variables were controlled for. A single value-added score for was then computed for each teacher. This score was meant to capture the difference between the actual and predicted grades for all the students in their course section. A high value-added score indicates that overall, the teacher instilled more learning than the model predicted. Therefore, the value-added score is a measure of the teacher’s contribution to learning in Calculus I. A similar procedure was used to compute the Calculus I instructors’ contribution to learning subsequent courses. The same non-teacher variables were used to predict grades in Calculus II and other follow-up courses, which were then compared to actual grades.

**FIGURE 2:**
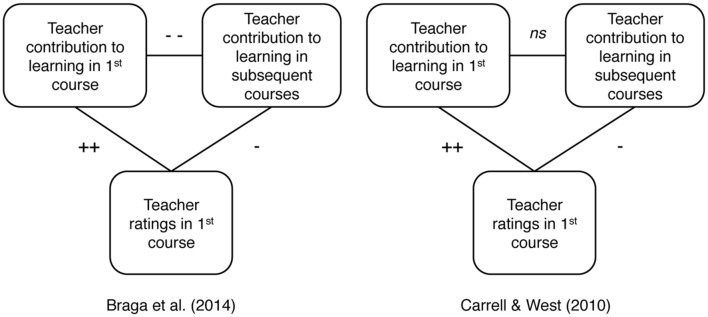
**Summary of the relationship between teacher ratings, value-added to first course and value-added to subsequent courses.** + Indicates positive and *p* < 0.05, ++ indicates positive and *p* < 0.01. - Indicates negative and *p* < 0.05, and -- indicates negative and *p* < 0.01. ns means not significantly different from 0.

Carrell and West (2010) found, consistent with prior studies, that teacher ratings were positively correlated with the teacher’s contribution to learning in first course. Subsequent course performance told a different story, however. The teachers who contributed more to learning as measured in follow-up courses had been given relatively *low* ratings in the first course. These teachers were also generally the more experienced teachers. In other words, getting low ratings in Calculus I was a sign that a teacher had made a relatively small contribution to learning as measured in Calculus I but a relatively large contribution to learning as measured in subsequent courses requiring calculus (**Figure 2**).

Braga et al. (2014) did a similar study of a cohort of approximately 1,200 first-year students who enrolled in 230 course-sections over the course of their 4 years at Bocconi University. This dataset had the same virtues of Carrell and West’s (2010) dataset (**Table 1**). Braga et al.’s (2014) data are a good complement to Carrell and West’s (2010) data because they ask the same question about a different type of student population and learning materials: Instead of a military academy in the United States the students attended a non-military school in Italy, and instead of calculus-based courses, the students in Braga et al.’s (2014) study took a fixed sequence of management, economics, or law courses.

Braga et al. (2014) found the same basic pattern of results as Carrell and West (2010). Teachers given higher ratings tended to have less experience. Receiving low ratings at the end of course 1 predicted that a teacher had (i) made a relatively small contribution to learning as measured at the end of course 1 and (ii) made a relatively large contribution to learning as measured in subsequent courses (**Figure 2**).

There is one other key finding from Braga et al.’s (2014) study. Intuitively, it seems obvious that a good teacher is a good teacher, regardless of whether knowledge is measured at the end of the teacher’s course or in subsequent courses. Braga et al.’s data belied this assumption. When a teacher made a relatively large contribution to knowledge in the first course, it could be reliably predicted that the teacher’s contribution to knowledge as measured in subsequent courses would be smaller than average. Carrell and West’s (2010) data showed a similar negative correlation, but it was not significant. (In one analysis, Carrell and West ranked teachers in terms of both contribution to course 1 and contribution to subsequent courses. The correlation between ranks was *r* = -0.68, but a significance level was not reported.) It is important to remember that these claims have to do with teacher contribution to learning, not individual student aptitude. Students who did better in course one also did better in subsequent courses, but individual student aptitude was controlled for in the value-added models (and by the fact that students were assigned to courses randomly).

It is difficult to interpret the strength of the correlations in **Figure 2** because of the complexity of the value added models, but three things seem clear. First, there is evidence from Carrell and West (2010), Braga et al. (2014), and other studies (Clayson, 2009), that when teacher contribution to learning is measured based on the teacher’s own course, teacher ratings are positively correlated with teacher effectiveness. Second, the data do not support the assumption that student ratings are an accurate way to estimate a teacher’s contribution to student knowledge after a course is over. Third, the data do not support the assumption that teacher contribution to learning in the teacher’s course is an accurate way to estimate a teacher’s contribution to student knowledge after a course is over.

## Why Did Better Teachers Get Lower Ratings?

Our conclusion is that better teachers got lower rating in the studies conducted by Carrell and West (2010) and Braga et al. (2014). In drawing, this conclusion we assume that the long-term goal of education is for knowledge to be accessible and useful after a course is over. Therefore, we consider the better teachers to be the ones who contribute the most to learning in subsequent courses. We refer to this kind of generalizable knowledge as “deep learning.”

Future research should examine how teachers’ decisions and classroom behavior affect ratings and deep learning. Until this research has been done, we can only speculate about why better teachers got lower ratings in these two studies. Our hypothesis is that better teachers may have created “desirable difficulties” for their students. Research shows that making learning more difficult has the same three effects that good teachers had in the studies reviewed here: it decreases short-term performance, decreases students’ subjective ratings of their own learning, and it simultaneously increased long-term learning and transfer (Schmidt and Bjork, 1992; Bjork, 1994; Rohrer and Pashler, 2010; Bjork and Bjork, 2011). For example, mixing different types of math problems on a problem set, rather than practicing one type of problem at a time, impairs performance on the problem set but enhances performance on a later test (e.g., Taylor and Rohrer, 2010; Rohrer et al., 2014). Most research on desirable difficulties has examined memory over a short period of time. Short-term performance typically refers to a test a few minutes after studying and long-term learning is usually measured within a week, whereas course evaluations take a full semester into account. However, the benefits of desirable difficulties have also been observed over the course of a semester (Rohrer et al., 2014).

Multiple studies have shown that learners rate desirable difficulties as counterproductive because their short-term performance suffers (e.g., Simon and Bjork, 2001; Kornell and Bjork, 2008). A similar effect seems to occur with teacher ratings: Making information fluent and easy to process can create an illusion of knowledge (Abrami et al., 1982; Carpenter et al., 2013), whereas classes that students perceive as more difficult tend to receive low ratings (Clayson and Haley, 1990; Marks, 2000; Paswan and Young, 2002; Centra, 2003).

It is not always clear which difficulties are desirable and which are not. Difficulties that have been shown to benefit classroom learning include frequent testing (e.g., Roediger and Karpicke, 2006; Lyle and Crawford, 2011) and interleaving problem types (e.g., Rohrer et al., 2014). However, not all difficulty is desirable; for example, poorly organized lectures make students’ lives difficult but are unlikely to enhance learning. **Table 3** lists teacher behaviors that seem likely to increase course difficulty and deep learning, but simultaneously decrease ratings. These behaviors are relevant even in situations where teaching to the test is not relevant, and their effects might be worth investigating in future research.

**Table 3:** Activities that seem likely to increase difficulty and long-term learning but decrease teacher ratings (based solely on the authors’ intuition).

Broaden the content being covered and include difficult concepts.
Focus on concepts that will be relevant beyond the current course.
Require students to struggle with the concepts they are learning (e.g., during lecture).
Give frequent quizzes.
Mix different kinds of problems together.
Assign relatively difficult problems in homework and class.
Do not circumscribe what students should study to prepare for their exams.
Give cumulative exams.

Of course, not every teaching decision that instills deep learning will decrease teacher ratings. In some circumstances students may be able to identifying effective teaching, even when it makes learning difficult. For example, Armbruster et al. (2009) reordered course content and added new lectures in an undergraduate introductory biology course to emphasize conceptual connections between topics. They also added daily in-class “clicker” quizzes and group problem solving activities. Final exam performance was significantly higher in semesters following the changes to the course than the semester prior to the changes. Furthermore, students gave higher overall ratings to the instructor after the course changed, despite also saying the course was more challenging. While there was no assessment of student performance in follow-up courses, these changes to the course seem likely to be desirable difficulties that enhanced deep learning, and not just performance on the end of semester exam.

Desirable difficulties have to do with the activities and processes learners are engaged in. It is possible that effective teachers also changed the content that they covered. In particular, perhaps these teachers broadened the curriculum and focused most on the most important, or difficult, concepts. Less effective teachers, by contrast, might have focused on preparing students to do well on the pre-determined set of questions that they knew would be on the test—that is, they might have been “teaching to the test” (Carrell and West, 2010; Braga et al., 2014).

Teaching to the test raises a potential limitation to our conclusions, because in a typical college course, if a teacher broadens the material, she can make the test correspond to the material she covered (i.e., “test to what she taught”). The existence of a pre-determined test might have meant the best teachers did not have the ability to adjust the test to fit their teaching. Thus, the results we have reviewed might have been different if there had been no common test to “teach to.” (In a typical college course there is no predetermined, unmodifiable test to teach to.) However, evidence against this potential concern already exists: Weinberg et al. (2009) examined courses in which teachers created their own tests and found that teacher ratings did not predict subsequent course performance when controlling grades in the first course. As mentioned earlier, Weinberg et al.’s study has its own limitations: it did not involve objective measures of learning and might have been affected by subject-selection effects. More research is needed about this potential objection.

## How Teacher Ratings Shape Teacher Incentives

Based on the negative relationship between ratings and deep learning, teacher ratings seem like a bad basis for decisions about hiring and promotion. However, we do not believe student ratings should be abolished, because ratings affect what they measure and the overall set of incentives they create might boost overall learning even if their correlation with learning is negative. As an analogy, imagine a teacher who is such a bad grader that when he grades papers, the correlation between grades and paper quality is slightly negative. One might argue that because these grades are unfair, it would be better if the teacher did not change the assignment save for one thing: no more grades. The problem, of course, is that the students would put far less effort into their papers—especially the students who were already not motivated—and the paper quality would drop precipitously. The measure of performance (student evaluations of teaching or, in the analogy, grades) might not be accurate or fair, but abolishing it could make performance (teaching, or in the analogy student papers) far worse. Whether abolishing ratings would be beneficial is an empirical question. To test this question would require a study that manipulated whether or not teachers were being rated and examined outcomes in terms of fairness to the teachers, teacher performance, and student learning. (A natural experiment of sorts already exists, in the sense that some schools put more emphasis on evaluations than others—and the former tend to have better teachers—but this difference is confounded with many other factors such as the proclivities of faculty who apply for such jobs.)

There are two reasons why student ratings might have overall net benefits for teachers. One is that they provide teachers with feedback on how they are seen by their students. The other is that they create a set of incentives that probably have a mix of positive and negative effects. On the positive side, they insure that teachers are prepared, organized, and responsive to students. We suspect that the average professor would put less time and effort into teaching if they were not concerned about student ratings (Love and Kotchen, 2010). As we have said, we think the positives might outweigh the negatives. On the negative side, the incentive to get good ratings can push teachers into making decisions that hurt student learning. We have already described some of these decisions (**Table 3**). Teachers should serve their students broccoli, but they tend to get higher ratings when they serve chocolate, and this is not just an analogy—one study showed that ratings increased when teachers literally served their students chocolate (Youmans and Jee, 2007). More generally, students tend to give high ratings when courses are easier or when they expect teachers to give them good grades, even if higher grades do not mean the students have learned more (Greenwald and Gillmore, 1997; Worthington, 2002; Isely and Singh, 2005; McPherson, 2006; Ewing, 2012). As a result, teacher ratings may be one factor contributing to grade inflation in recent decades (Krautmann and Sander, 1999; Eiszler, 2002; Johnson, 2003; Love and Kotchen, 2010).

It is not just professors who have incentives to ensure that teacher ratings are high. Colleges and universities have strong incentives to boost the satisfaction, and perhaps charitable giving, of future alumni. Student ratings may be a perfect way to identify teachers who accomplish this goal, that is, teachers whose students enjoyed their courses and *think* they have learned a lot. (There is also an incentive for schools to insure that their students get a good education so they can succeed in their lives and careers, but it is infinitely easier to measure student evaluations than it is to parse out a single professor’s impact on their students’ lives twenty years later.)

## Conclusion

Two recent studies found that when learning was measured as performance in subsequent related courses (i.e., when deep learning was measured), teachers who made relatively large contributions to student learning received relatively low teacher ratings (Carrell and West, 2010; Braga et al., 2014). If a college’s main goal is to instill deep, long-term learning, then teacher ratings have serious limitations.

Just as it is misguided to assume that ratings have any obvious relationship with student learning, it is also misguided to assume that end-of-semester test performance is a valid measure of deep learning. Teacher effectiveness as measured by students’ performance on end-of-semester exams was negatively correlated with teacher effectiveness as measured in subsequent courses (Braga et al., 2014). If these results generalize, it would suggest that standardized test performance can be at odds with durable, flexible knowledge (though it seems safe to assume that they match up well in some situations). This would be a troubling conclusion for at least two reasons. First, most measures of learning focus on the material learned during the preceding semester or year. Such measures may fail to capture deep learning, or even create an impression opposite to the truth. Second, primary and secondary school teachers are often incentivized, or required, to teach to tests. This requirement might actually undermine deep learning.

We do not recommend abandoning teacher ratings, at least not in the absence of controlled experiments comparing teachers who are being rated to teachers who are not. Teacher ratings provide incentives for teachers to invest effort in their teaching. However, these incentives might also harm teaching in some ways (**Table 3**), and we recommend that future research should investigate ways of eliciting student ratings that correlate better with deep learning.

How should teacher effectiveness be assessed? The student perspective is important, but students do not necessarily have the expertise to recognize good teaching. Their reports reflect their experiences, including whether they enjoyed the class, whether the instructor helped them appreciate the material, and whether the instructor made them more likely to take a related follow-up course. We think that these factors should be taken into account when assessing how good a teacher is. But it is also important to take into account how much the students learned, and students are simply not in a position to make accurate judgments about their learning. In the end, student ratings bear more than a passing similarity to a popularity contest.

We recommend that student ratings should be combined with two additional sources of data, both of which would require a significant investment of resources. First, teachers should receive more “coaching” from expert teachers, who can assess and rate them and also make suggestions (Murray, 1983; Braskamp and Ory, 1994; Paulsen, 2002; Berk, 2005). For one example of what a more holistic faculty review system could look like, consider the University of California, Berkeley’s statistics department (Stark and Freishtat, 2014). Second, where possible, steps should be taken to measure deep knowledge by examining teacher contribution to knowledge in a fair and objective way after students have completed a professor’s course.

## Author Contributions

The idea originated with NK. NK and HH did the writing together. HH did the majority of the literature review.

## Conflict of Interest Statement

The authors declare that the research was conducted in the absence of any commercial or financial relationships that could be construed as a potential conflict of interest.
